# Integrative serum metabolite prioritization and functional screening identify N-acetyl-L-glutamine as a protective candidate in premature ovarian insufficiency

**DOI:** 10.3389/fgene.2026.1827858

**Published:** 2026-07-02

**Authors:** Xinyue Zhang, Chen Chen, Xiaolan Zhu

**Affiliations:** Reproductive Medicine Center, The Fourth Affiliated Hospital of Jiangsu University, Zhenjiang, Jiangsu, China

**Keywords:** genetics, infertility, metabolomics, premature ovarian insufficiency, target prioritization

## Abstract

**Background:**

Premature ovarian insufficiency (POI) is a major cause of female infertility and is increasingly associated with systemic metabolic dysregulation. However, whether circulating metabolic alterations contribute causally to POI development or primarily arise as secondary consequences of ovarian failure remains unclear. In this study, bidirectional Mendelian randomization (MR), cell-based screening, and exploratory target-prioritization analyses were integrated to identify POI-related metabolites and functionally relevant candidates.

**Methods:**

Two-sample MR was performed using genome-wide association studies (GWAS) summary data for 1,400 serum metabolites/metabolite ratios and POI. After instrumental variable filtering and harmonization, 1,352 exposures with valid inverse-variance weighted (IVW) estimates were retained for forward MR and multiple-testing correction. Both Benjamini–Hochberg false discovery rate (FDR) and Bonferroni correction were applied. Reverse MR was then conducted as a secondary directionality analysis to assess whether genetic liability to POI was also associated with circulating metabolic alterations. Experimentally tractable metabolites were screened in cyclophosphamide (CTX)-injured KGN cells using CCK-8 assays and Western blotting. For the prioritized metabolite, further functional validation was performed using SA-β-gal staining, ROS detection, and JC-1 assays. Proteome-wide MR, colocalization analysis, summary-data-based MR (SMR), drug prediction, and molecular docking were subsequently conducted as exploratory downstream analyses.

**Results:**

Among the 1,352 analysable exposures, 54 showed nominal associations with POI at *P*_IVW_ < 0.05. After FDR and Bonferroni correction, sphinganine-1-phosphate remained the only metabolite that reached multiple-testing-corrected significance and was positively associated with POI risk, suggesting that it may represent a risk-associated metabolic candidate. Reverse MR identified exploratory POI-to-metabolite associations for six metabolites, indicating that genetic liability to POI may also be linked to systemic metabolic alterations. The experimental screening aimed to identify protective metabolites; therefore, N-acetyl-L-glutamine was prioritized from nominal inverse MR signals on the basis of its protective direction, glutamine-related identity, biological plausibility, and feasibility for cell-based assays. Among the five screened metabolites, N-acetyl-L-glutamine had the most consistent protective effect in CTX-injured KGN cells, attenuating p21 and p53 upregulation, reducing the number of SA-β-gal-positive cells and the accumulation of ROS, and partially restoring the mitochondrial membrane potential. Downstream analyses identified LILRB1 as an exploratory candidate protein linked to N-acetyl-L-glutamine levels that warrants further investigation, and cianidanol as a computational lead requiring functional validation.

**Conclusion:**

This study identifies N-acetyl-L-glutamine as a biologically plausible and experimentally supported protective metabolite candidate that attenuates CTX-induced senescence, oxidative stress, and mitochondrial dysfunction in granulosa-like cells. By integrating metabolome-wide MR with bidirectional analyses, our findings support a metabolite-centred framework for investigating POI-related metabolic vulnerability and oncofertility-related ovarian injury. Sphinganine-1-phosphate emerged as a multiple-testing-corrected risk-associated metabolite, whereas LILRB1 and cianidanol generated exploratory hypotheses for future mechanistic and pharmacological studies.

## 1 Introduction

Premature ovarian insufficiency (POI) refers to the loss or marked impairment of ovarian function before 40 years of age and affects approximately 1% of women of reproductive age (“ESHRE Guideline,” 2016). Clinically, POI is characterized by infertility, elevated gonadotropin levels, and hypoestrogenism (Wang et al., 2022). Patients often experience vasomotor symptoms, sleep disturbances, reduced libido, genital atrophy, dyspareunia, and mood changes, all of which can substantially impair quality of life(Nelson, 2009). Although hormone replacement therapy (HRT) can alleviate symptoms related to oestrogen deficiency (Sullivan et al., 2016), it does not restore ovarian reserve or directly modify the upstream pathological processes that drive POI (Ghahremani-Nasab et al., 2020). Because the causes of POI remain incompletely understood (Goswami & Conway, 2005), identifying nonhormonal and disease-relevant biological candidates remains an important unmet need.

With the rapid development of omics technologies, metabolomics has become an important approach for investigating disease biology. By capturing intermediate metabolites and pathway-level alterations, metabolomics provides a direct window into dynamic biological processes (Johnson et al., 2016; Joyce & Palsson, 2006). Large-scale metabolite genome-wide association studies (GWASs) have further advanced this field by identifying genetic loci associated with circulating metabolites and metabolic regulation (Yin et al., 2022). Recent metabolite GWAS resources have enabled the investigation of genetically determined metabolites (GDMs), which may help link systemic metabolic variation to disease susceptibility (CARDIoGRAM et al., 2011; Gieger et al., 2008; Kettunen et al., 2012; The Multiple Tissue Human Expression Resource Muther Consortium et al., 2014). Several observational studies have suggested that metabolic abnormalities are associated with ovarian ageing or POI. For example, Moslehi et al. reported that 14 serum metabolites, including phosphate, N-acetyl-D-glucosamine, and proline, were associated with a rapid decrease in anti-Müllerian hormone levels and reflected ovarian ageing(Moslehi et al., 2021). Liu et al. reported that the levels of several serum proteins were altered in patients with POI, whereas Zhou et al. reported that plasma metabolites were differentially expressed in patients with POI(Liu et al., 2020; Zhou et al., 2023). However, these observational findings cannot be used to determine whether metabolic alterations contribute to ovarian dysfunction or mainly reflect secondary consequences of follicular depletion, hypoestrogenism, or systemic changes after POI onset.

Mendelian randomization (MR) is a genetic epidemiologic approach that uses inherited variants as instrumental variables to evaluate potential causal relationships between exposures and outcomes(Smith, 2004). Compared with conventional observational analyses, MR can reduce bias from confounding and reverse causation because alleles are randomly allocated at conception(Davey Smith & Hemani, 2014; Lawlor et al., 2008). In the context of POI metabolomics, bidirectional MR provides a strategy to examine both metabolite-to-POI and POI-to-metabolite directions, thereby addressing whether circulating metabolic alterations are more likely to act as upstream contributors, downstream consequences, or part of a bidirectional metabolic disturbance. In the present study, we integrated genetically informed metabolite prioritization with cell-based functional screening. We first assessed the associations between serum metabolites and POI using two-sample and bidirectional MR. We then evaluated experimentally tractable candidates in CTX-injured granulosa-like cells to identify metabolites with potential protective activity against POI-related cellular injury, followed by exploration of downstream proteins and compound prioritization.

## 2 Materials and methods

### 2.1 Genome-Wide Association Study of Serum Metabolites

Genetic association data for circulating metabolites were obtained from the study by Chen et al. (2023), which conducted genome-wide association analyses using both metabolites and metabolite ratios as phenotypes. This dataset included 8,299 adults from the Canadian Longitudinal Study on Aging (CLSA) cohort. Metabolite identification, relative quantification, data reduction, and quality control were performed using the standardized Metabolon platform (https://www.metabolon.com/) (Ryals et al., 2007). In total, 1,091 metabolites and 309 metabolite ratios were retained for the final GWAS analysis. These metabolites were classified into eight broad categories: amino acids, carbohydrates, cofactors and vitamins, energy, lipids, nucleotides, peptides, and xenobiotics. Notably, 241 metabolites (22%) were annotated as “unknown,” indicating that their precise chemical identities have not yet been fully resolved. These metabolites were retained because they may still contain biologically informative signals (Krumsiek et al., 2012). After genotyping, imputation, and genetic ancestry determination by the CLSA group (Forgetta et al., 2022), variants with poor imputation quality were excluded. The final GWAS meta-analysis included approximately 15.4 million SNPs with a minor allele frequency (MAF) > 0.1%, an imputation quality score > 0.3, and missingness < 0.1.

### 2.2 Genome-wide association study of premature ovarian insufficiency

Summary statistics for POI were obtained from FinnGen Consortium data release R11 (https://r11.finngen.fi/) (Kurki et al., 2023), which included 599 cases and 241,998 controls among adult Finnish women. FinnGen integrates genotype data from Finnish biobanks with nationwide health registry information. Study endpoints were defined using International Classification of Diseases codes, and the original analyses were adjusted for sex, age, genotyping batch, and the first ten principal components (Kurki et al., 2023). Because only publicly available summary-level data were used, no additional ethical approval or informed consent was required for the present analysis. Given that the metabolite GWAS was derived from the CLSA cohort and the POI GWAS was obtained from FinnGen, substantial sample overlap was unlikely. Both datasets were predominantly of European ancestry; however, potential differences between the Canadian cohort and the Finnish genetic isolate were considered when interpreting the transferability of the findings. Because the POI GWAS included 599 cases, the statistical power to detect modest metabolite–POI associations was limited. Therefore, nominal MR associations were interpreted cautiously and were not considered definitive causal evidence without multiple-testing correction or functional support.

### 2.3 Genetic instrumental variables for 1400 metabolites and metabolite ratios

To satisfy the core assumptions of MR, we applied a standardized procedure to select genetic instrumental variables for all 1,400 metabolites and metabolite ratios. Because the sample sizes of available metabolite GWAS datasets are generally smaller than those of large-scale disease GWASs, applying the conventional genome-wide significance threshold of 5 × 10^-8^ would have left many metabolites with few or no eligible instruments. Therefore, to balance instrument availability and instrument strength in this exploratory metabolome-wide MR analysis, we adopted an instrument-selection threshold of *P* < 1 × 10^-5^.

SNPs associated with each metabolite at *P* < 1 × 10^-5^ were extracted and subjected to linkage disequilibrium clumping using the European 1000 Genomes reference panel to obtain independent variants (*r* ^*2*^ < 0.001). Instrument strength was assessed by calculating the proportion of variance explained (*R* ^*2*^) and the F statistic(Pierce et al., 2011). To reduce the risk of weak-instrument bias, only instruments with F statistics > 10 were retained for subsequent MR analyses(Brion et al., 2013). Detailed R^2^ values and F statistics for the selected instruments are provided in Supplementary Table S2.

### 2.4 MR harmonization and quality control

Exposure and outcome summary statistics were harmonized before MR analysis to ensure that SNP effects corresponded to the same effect allele. Palindromic SNPs with ambiguous allele frequencies and SNPs with incompatible alleles were removed during harmonization. SNPs were further excluded if they were unavailable in the outcome dataset, failed harmonization, were removed during LD clumping, or showed weak instrument strength (F ≤ 10). Harmonized SNP-level information, including exposure and outcome alleles, palindromic and ambiguous SNP flags, MR-retention status, SNP-level Steiger directionality information, R^2^ values, and F statistics, is provided in Supplementary Table S2. In addition, metabolite-level Steiger directionality assessment was performed for nominally associated exposures and is reported in Supplementary Table S4.

For Steiger directionality assessment, SNP-exposure correlations were estimated for quantitative metabolite exposures, whereas SNP-outcome correlations for the binary POI outcome were estimated using log-odds ratios, effect allele frequencies, and the number of POI cases and controls. Because SNP-level outcome sample sizes were not available in the FinnGen summary statistics, the total FinnGen POI sample size was assigned as 242,597, including 599 cases and 241,998 controls. Because POI is a binary outcome and variance-explained estimates may be influenced by measurement scale and case-control structure, Steiger directionality assessment was used as sensitivity evidence rather than as the sole exclusion criterion. Metabolites with inconsistent or unsupported Steiger directionality were interpreted cautiously.

For metabolites showing nominal associations with POI, additional sensitivity analyses were summarized at the metabolite level. Heterogeneity was assessed using Cochran’s Q statistic, and directional horizontal pleiotropy was evaluated using the MR-Egger intercept test. MR-PRESSO global tests were applied, where applicable, to assess outlier-related pleiotropy. Leave-one-out analyses were performed to determine whether each association was driven by a single SNP. These metabolite-level sensitivity results, together with metabolite-level Steiger directionality summaries, are provided in Supplementary Table S4. When interpreting the MR findings, metabolites showing evidence of heterogeneity, directional pleiotropy, potential single-SNP influence, or unsupported Steiger directionality were considered with caution.

### 2.5 Multiple-testing correction

Of the 1,400 metabolites and metabolite ratios initially considered, exposures with insufficient valid instrumental variables after SNP selection, LD clumping, harmonization, and weak-instrument filtering were excluded before MR estimation. Consequently, 1,352 exposures with available inverse-variance weighted (IVW) MR estimates were included in the forward MR analysis and multiple-testing correction.

Nominal significance was defined as *P*_IVW_ < 0.05. To control for multiple testing across all analysable exposures, Benjamini–Hochberg false discovery rate (FDR) correction and Bonferroni correction were applied to the IVW *P* values of the 1,352 exposures using the p.adjust() function in R. For the Benjamini–Hochberg procedure, raw *P* values were ranked from smallest to largest, and the FDR-adjusted *P* value was calculated as:

$$P_{FDR,\left(i\right)}=\min_{j\geq i}\left(mP_{\left(j\right)}/j,1\right)$$

where P_(j)_ represents the *j*th smallest raw IVW *P* value and *m* = 1,352 represents the number of analysable exposures with valid MR estimates. Bonferroni-adjusted *P* values were calculated as:

$$P_{Bonferroni}=\min\left(P_{raw}\times m,1\right)$$

corresponding to a Bonferroni significance threshold of:

$$0.05/1,352=3.70\times 10^{-5}$$

Metabolites with FDR-adjusted *P* values < 0.05 were considered FDR-supported associations. Metabolites with *P*_IVW_ < 0.05 but FDR-adjusted *P* ≥ 0.05 were considered nominal associations. The complete MR results for all 1,352 analysable exposures, including raw IVW *P* values, FDR-adjusted *P* values, and Bonferroni-adjusted *P* values, are provided in Supplementary Table S3.

### 2.6 Cell culture and *in vitro* injury model

KGN cells were obtained from Shanghai Jihe Biotechnology Co., Ltd. (China). The cells were cultured in F12/DMEM medium (Gibco, USA) supplemented with 10% fetal bovine serum (Gibco, USA) and 1% penicillin–streptomycin (HyClone, USA). Cells were maintained at 37 °C in a humidified incubator with 5% CO_2_. All experiments were performed using cells between passages 3 and 5. To induce cellular senescence, KGN cells were seeded into six-well plates, allowed to adhere for 12 h, and then exposed to cyclophosphamide (CTX, 100 μM) for 24 h. The CTX-injured KGN cell model was used as a preliminary granulosa-like cell injury model to evaluate whether prioritized metabolites could modulate POI-related cellular phenotypes, including senescence, oxidative stress, and mitochondrial dysfunction. This model was not intended to directly recapitulate genetic susceptibility to POI, but rather to provide a functional screening system for shared injury-related pathways. KGN cells were selected because they retain several granulosa-cell-like features and are widely used in studies of ovarian function; however, their tumor-derived origin was considered when interpreting senescence-related readouts.

### 2.7 Metabolite treatment and candidate screening

Candidate metabolites for cell-based screening were prioritized from the MR-nominated metabolite pool using a stepwise strategy that considered statistical evidence, association direction, chemical interpretability, biological relevance, metabolic-class representativeness, and experimental feasibility. This prioritization strategy incorporated investigator judgment and was not intended to provide an objective ranking of all nominally associated metabolites, but rather to generate experimentally tractable hypotheses for functional screening. Because the experimental screen aimed to identify metabolites with potential protective activity against granulosa-cell injury, metabolite ratios and metabolites annotated as unknown were not prioritized for direct cell-based validation. Metabolite ratios are less suitable for supplementation-based cell treatment, whereas unknown metabolites have limited chemical interpretability.

Among the 54 nominal forward MR associations, we first distinguished multiple-testing-corrected findings from nominal associations. Sphinganine-1-phosphate was the only metabolite that survived both FDR and Bonferroni correction, but its genetically predicted level was positively associated with POI risk. Therefore, it was interpreted as a corrected-significant risk-associated metabolite rather than a protective supplementation candidate. After excluding metabolite ratios and unknown metabolites, 18 chemically defined individual metabolites with nominal inverse associations with POI risk remained and were considered as the protective candidate pool for functional prioritization.

These 18 metabolites were further evaluated according to MR direction, chemical interpretability, biological or pathway rationale, practical feasibility for *in vitro* treatment, and metabolic-class representativeness. Based on this strategy, N-acetyl-L-glutamine, 3-formylindole, DHEAS, 4-methyl-2-oxopentanoate, and sucrose were selected for preliminary functional screening. N-acetyl-L-glutamine was prioritized as the primary functional candidate because it combined a nominal inverse MR direction, clear glutamine-related biochemical identity, biological relevance to redox balance and mitochondrial homeostasis, and feasibility for low-dose *in vitro* treatment. The other four metabolites were included as comparator compounds representing indole-related, steroid/endocrine-related, branched-chain amino-acid-related, and carbohydrate-related metabolic contexts. This pathway-diverse design allowed preliminary comparison across different metabolic categories, while available literature provided biological context for selected categories, including indole-related oxidative/inflammatory regulation (Cao et al., 2024) and branched-chain amino-acid-related metabolism (Lim et al., 2025).

To determine suitable working concentrations under injury conditions, KGN cells were exposed to CTX together with concentration gradients of each candidate metabolite, and cell viability was assessed using CCK-8 assays. CTX + DMSO-treated cells were used as the vehicle-treated injury control. The concentration ranges were selected based on compound-specific solubility, preliminary viability testing, and available literature on related compounds or biological activities (Cao et al., 2024; Jiang et al., 2023; Kim et al., 2015; Lim et al., 2025; Zhang et al., 2015). Final working concentrations were determined according to viability responses, solubility, and feasibility for subsequent protein-based screening.

The upper concentrations of sucrose and 4-methyl-2-oxopentanoate were used only in the initial viability screening to define concentration–response profiles. Because osmolarity was not directly measured, potential osmotic effects at the highest screening concentrations could not be fully excluded. Therefore, results obtained at these high concentrations were interpreted with caution and used only to guide the selection of working concentrations.

### 2.8 Cell counting kit-8 assay

KGN cells were seeded in 96-well plates at a density of 4 × 10^3^ cells per well in 100 μL of complete medium. After 24 h, the cells were treated with CTX together with the corresponding vehicle control or concentration gradients of the indicated metabolites, which were prepared in appropriate solvents according to their solubility. CTX + DMSO-treated cells were used as the vehicle-treated injury control when DMSO was used. The final vehicle concentration was kept consistent across all groups. After 48 h of exposure, the medium was removed, and the cells were washed with phosphate-buffered saline (PBS). Fresh medium containing 10% Cell Counting Kit-8 reagent (Ab228554, Abcam) was then added to each well. After incubation for 2 h at 37 °C in the dark, absorbance at 450 nm was measured using a SUNRISE microplate reader. Cell viability was normalized to that of the untreated control group.

### 2.9 Western blotting

Cells were lysed in RIPA buffer (Solarbio, Beijing, China) supplemented with a protease inhibitor cocktail (Solarbio, Beijing, China). Protein concentrations were determined, and equal amounts of protein were mixed with 5× loading buffer (Beyotime, Shanghai, China) and denatured by boiling for 5 min. Protein samples were separated by 8% or 10% SDS–PAGE and transferred onto PVDF membranes (Millipore, USA). After blocking with 5% nonfat milk for 2 h at room temperature, the membranes were incubated overnight at 4 °C with primary antibodies against p53 (Proteintech, China, 1:5000), p21 (Proteintech, China, 1:2000), and β-actin (Proteintech, China, 1:20000). The membranes were then incubated with the corresponding secondary antibody (Biosharp, China, 1:10000) for 1.5 h at room temperature. Protein bands were visualized using ECL reagent (Vazyme, Nanjing, China) and quantified using ImageJ software.

### 2.10 SA-β-galactosidase staining

Cellular senescence was assessed using a Senescence β-Galactosidase Staining Kit (Beyotime, Shanghai, China) according to the manufacturer’s instructions. Briefly, cells cultured in six-well plates were fixed with 1 mL of fixative per well for 15 min at room temperature and washed twice with PBS. Freshly prepared SA-β-gal staining solution was then added, and the cells were incubated overnight. Blue-stained cells were identified as senescent cells under a light microscope (Leica Microsystems, Mannheim, Germany). The percentage of SA-β-gal-positive cells was calculated from randomly selected microscopic fields across three independent experiments.

### 2.11 ROS assay

Intracellular ROS levels were measured using a Reactive Oxygen Species Assay Kit (Beyotime, Shanghai, China). KGN cells were incubated with DCFH-DA for 30 min at 37 °C in the dark and then washed with PBS. Nuclei were counterstained with Hoechst 33342 (Beyotime, Shanghai, China) for 10 min at room temperature in the dark. Fluorescence images were captured immediately using a fluorescence microscope (Leica Microsystems, Mannheim, Germany), and fluorescence intensity was quantified using ImageJ software.

### 2.12 Mitochondrial membrane potential (ΔΨm) assay

Mitochondrial membrane potential was evaluated using a JC-1 Mitochondrial Membrane Potential Assay Kit (Beyotime, Shanghai, China). Cells were incubated with JC-1 working solution at 1 mg/L for 20 min at 37 °C, washed with PBS, and immediately observed under a fluorescence microscope (Leica Microsystems, Mannheim, Germany). Changes in mitochondrial membrane potential were assessed by calculating the red-to-green fluorescence ratio using ImageJ software.

### 2.13 Genome-Wide Association Study of pQTL

Druggable genes were defined as genes encoding proteins that can potentially be modulated by drug-like small molecules, based on sequence or structural similarity to known drug targets(Finan et al., 2017). According to Finan et al., 4,479 druggable genes were identified, including 1,427 genes encoding approved or clinical-stage drug targets, 682 genes encoding proteins that bind known drug molecules or resemble approved drug targets, and 2,370 genes belonging to major druggable gene families or showing distant similarity to approved targets(Finan et al., 2017). This resource provided a broad set of potential targets for downstream analyses (Supplementary Table S1).

Blood cis-eQTL data were available for 2,525 of these 4,479 druggable genes from the eQTLGen Consortium(Võsa et al., 2021), which integrates 37 datasets comprising 31,684 individuals, predominantly of European ancestry. These data capture genetic variants located within 1 Mb of each gene that are associated with blood gene expression, and all included variants had a MAF > 0.01.

pQTL summary statistics were obtained from the FinnGen Proteomics Summary Statistics dataset(Kurki et al., 2023). A total of 2,925 proteins were available, and pQTLs corresponding to druggable genes were extracted to further investigate protein–metabolite relationships. After intersection with druggable genes and instrumental-variable filtering, 610 plasma proteins were retained for protein–metabolite MR.

These pQTL data were used as genetic instruments for plasma proteins in downstream protein–metabolite MR analysis, in which N-acetyl-L-glutamine levels were treated as the outcome. Blood cis-eQTL data from eQTLGen were used for gene-expression-based SMR and colocalization analyses where applicable.

### 2.14 Colocalization

For candidate protein signals identified in the protein–metabolite MR analysis, colocalization analysis was performed using the R package “coloc” version 5.1.0.1. The default prior probabilities were set to *P*1 = 1.0 × 10^-4^, *P*2 = 1.0 × 10^-4^, and *P*12 = 1.0 × 10^-5^, representing the probabilities that a SNP is associated with the QTL signal, the outcome, or both, respectively. Posterior probabilities were estimated for five hypotheses: PPH0, no association with either trait; PPH1, association with the QTL signal only; PPH2, association with the outcome only; PPH3, association with both traits but through distinct causal variants; and PPH4, association with both traits through a shared causal variant. A PPH4 value ≥ 0.75 was used as a predefined threshold for colocalization evaluation. The SNP most strongly associated with the exposure was used as the reference variant, and variants within ±500 kb were included in the analysis. Because colocalization results can be sensitive to variant coverage, signals based on only a small number of SNPs were interpreted with caution. Colocalization findings were not interpreted solely according to the PP.H4 threshold; the number of variants supporting each signal was also considered when evaluating robustness.

### 2.15 Summary-data-based MR

Summary-data-based Mendelian randomization (SMR) extends the MR framework by integrating GWAS and QTL summary statistics to prioritize putative causal genes underlying GWAS signals. When combined with the heterogeneity in dependent instruments (HEIDI) test, SMR helps distinguish pleiotropic or potentially causal effects from associations driven by linkage disequilibrium. In this study, SMR analysis was performed using SMR software (Windows version 1.0.3) with default settings (https://yanglab.westlake.edu.cn/software/smr/#Overview).

### 2.16 Candidate drug prediction

To evaluate whether candidate genes had potential pharmacological connections, candidate compounds were predicted using the Drug Signatures Database (DSigDB, http://dsigdb.tanlab.org/DSigDBv1.0/) (Yoo et al., 2015). DSigDB contains 22,527 gene sets and 17,389 distinct compounds spanning 19,531 genes, enabling systematic matching between genes and known chemical perturbagens. Target genes identified in this study were uploaded to DSigDB to predict candidate compounds with potential medicinal relevance. Candidate drug prediction was performed as an exploratory analysis. Predicted compounds were not considered validated therapeutic candidates unless supported by experimental functional evidence. Adjusted P values from DSigDB were reported, and compounds that did not pass multiple-testing correction were interpreted as computational leads only.

### 2.17 Molecular docking

Molecular docking was performed to predict the binding mode between a small-molecule ligand and its protein receptor. A semi-flexible docking strategy was adopted in this study. Docking between cianidanol (PubChem CID: 9064) and LILRB1 (UniProt ID: Q8NHL6) was conducted using AutoDock Vina 1.1.2(Trott & Olson, 2010). The protein structure was downloaded from the AlphaFold Protein Structure Database. Protein preprocessing was performed in PyMOL 2.4 and included the removal of water molecules and excess ligands, followed by the addition of hydrogen atoms. The ligand structure was energy-minimized in ChemDraw 20.0, and PDBQT files were generated using AutoDock Tools 1.5.6. The docking box was set to cover the entire protein, and all other parameters were kept at their default settings. Nine docking conformations were generated, and the conformation with the lowest binding energy and highest clustering frequency was considered the most probable binding mode. Docking results were visualized using PyMOL 2.4 and Discovery Studio 2019. The inhibition constant (*K*i) was estimated from the docking binding energy using the thermodynamic relationship *K*i = exp(Δ*G*/RT).

### 2.18 Statistical Analysis

Two-sample MR analyses of serum metabolites and POI were primarily performed using the inverse-variance weighted (IVW) method (Burgess et al., 2013). Under the assumption that all genetic instruments are valid, IVW provides efficient and consistent causal estimates. Conceptually, the IVW estimate can be interpreted as a regression of SNP–outcome effects on SNP–exposure effects with the intercept constrained to zero. For uncorrected MR analyses, *P* < 0.05 was considered nominally significant. In the metabolome-wide forward MR analysis, FDR-adjusted *P* < 0.05 was considered multiple-testing-corrected significance, and Bonferroni correction was applied as a more conservative threshold.

Because horizontal pleiotropy can bias MR results, several sensitivity analyses were also performed. These included the weighted median method, which remains valid when fewer than 50% of the instruments are invalid (Bowden, Davey Smith, et al., 2016); MR-Egger regression, which can detect and partially account for directional pleiotropy (Bowden, Del Greco M., et al., 2016); and MR-PRESSO, which identifies and removes outlier variants contributing to horizontal pleiotropy (Verbanck et al., 2018). The MR-PRESSO global test was additionally used to assess overall pleiotropy. MR data harmonization and Steiger directionality assessment were performed using the R package “TwoSampleMR”(Hemani et al., 2018), while MR estimates and sensitivity analyses were performed using “MendelianRandomization” and MR-PRESSO where applicable (Verbanck et al., 2018).

Reverse MR was performed as a secondary directionality analysis using POI as the exposure and nominally associated metabolites as outcomes. This analysis was conducted to explore whether genetic liability to POI might also be linked to circulating metabolic alterations. Reverse MR findings were therefore interpreted as exploratory directionality signals rather than definitive evidence of reverse causality.

To explore candidate proteins genetically associated with N-acetyl-L-glutamine levels, an additional two-sample MR analysis was performed using plasma proteins as exposures and N-acetyl-L-glutamine as the outcome. This analysis was therefore designed to identify protein–metabolite associations, rather than to determine whether N-acetyl-L-glutamine directly regulates these proteins. For proteins instrumented by a single variant, MR estimates were obtained using the Wald ratio. For proteins with more than one instrument, IVW was applied. Heterogeneity was assessed using the Q statistic. Additional sensitivity analyses included simple mode, weighted mode, weighted median, and MR-Egger methods. MR-Egger estimates were interpreted cautiously, particularly when the intercept suggested potential horizontal pleiotropy.

For cell-based experiments, data are presented as the mean ± SD from at least three independent experiments unless otherwise stated. Comparisons between two groups were performed using Student’s *t*-test, whereas comparisons among multiple groups were performed using one-way ANOVA followed by Tukey’s *post hoc* test. Statistical analyses for cell experiments were performed using GraphPad Prism or R, and *P* < 0.05 was considered statistically significant.

## 3 Results

### 3.1 Study design

**FIGURE 1:**
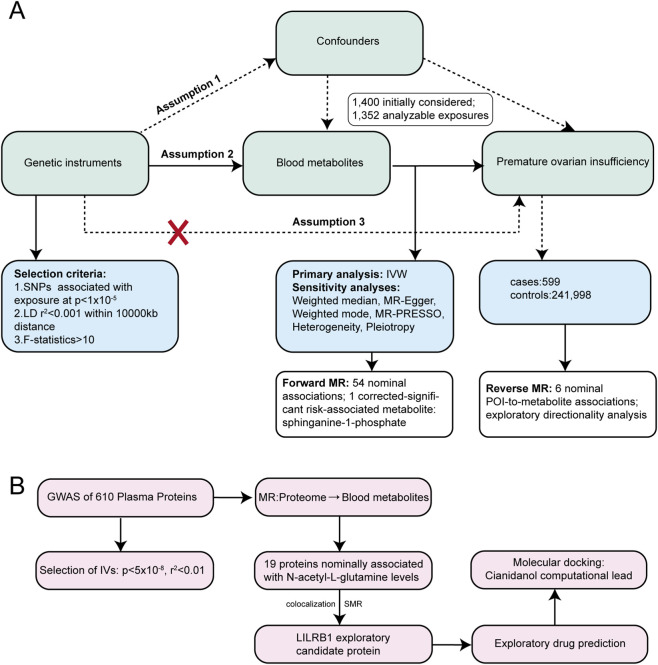
Overview of the study workflow. **(A)** Bidirectional MR analysis of serum metabolites and premature ovarian insufficiency, followed by functional screening in CTX-injured KGN cells. **(B)** Exploratory protein–metabolite MR, colocalization/SMR, drug prediction, and molecular docking analyses for N-acetyl-L-glutamine-related candidates.

All datasets used in this study were publicly available and had received prior ethical approval. We first evaluated the associations between circulating metabolites and POI using a genetically informed MR framework. The study design followed the three core assumptions of MR: genetic instruments should be strongly associated with the exposure, independent of potential confounders, and influence the outcome only through the exposure of interest. We then integrated metabolome-wide MR, reverse MR, cell-based functional screening, protein–metabolite MR, colocalization analysis, SMR, drug prediction, and molecular docking to prioritize POI-related metabolites and explore downstream candidates. The overall workflow is shown in Figure 1.

### 3.2 Genetic instruments for serum metabolites

Instrumental variables were selected for the 1,400 serum metabolites and metabolite ratios using the procedure described above. After SNP selection, LD clumping, harmonization, and weak-instrument filtering, 1,352 exposures retained sufficient valid instruments and yielded IVW MR estimates. The F statistics of the retained instruments exceeded the empirical threshold of 10, with a minimum value of 19.51, suggesting that weak-instrument bias was unlikely to be substantial. Harmonized SNP-level information, including allele alignment, palindromic and ambiguous SNP flags, MR-retention status, SNP-level Steiger directionality information, R^2^ values, and F statistics, is provided in Supplementary Table S2. To facilitate exposure-level interpretation, metabolite-level Steiger directionality summaries were further generated for the 54 nominally associated exposures and are provided in Supplementary Table S4.

### 3.3 Forward MR analysis of serum metabolites and POI

**FIGURE 2:**
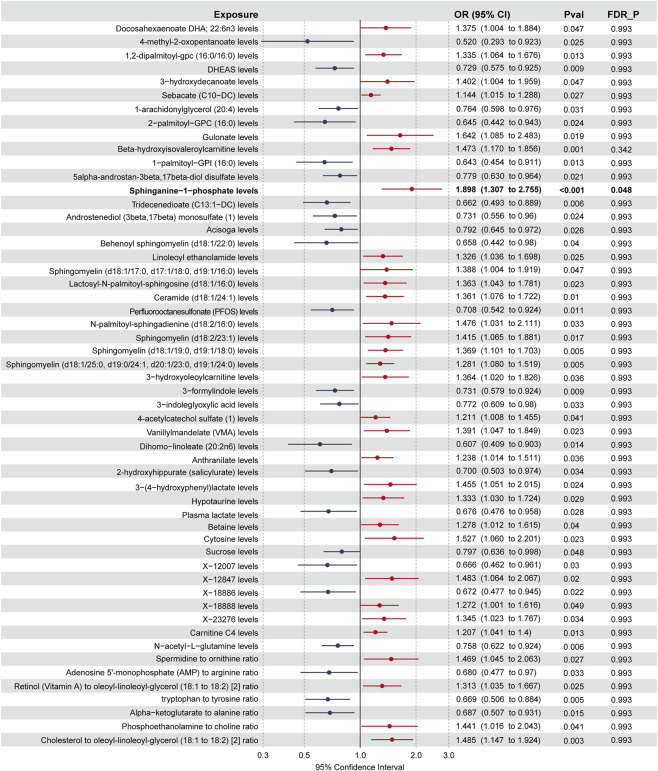
Forward MR associations between serum metabolites and POI risk using the IVW method. The forest plot shows exposures reaching nominal significance at P_IVW_<0.05. Sphinganine-1-phosphate remained significant after FDR and Bonferroni correction and was interpreted as a corrected-significant risk-associated metabolite candidate. The remaining exposures were considered nominal MR signals.

When IVW was used as the primary MR method, 54 of the 1,352 analysable exposures showed nominal associations with POI at *P*_IVW_ < 0.05, including 47 individual metabolites and 7 metabolite ratios (Figure 2 and Supplementary Table S3). After Benjamini–Hochberg FDR correction and Bonferroni correction across all 1,352 analysable exposures, sphinganine-1-phosphate remained the only exposure that reached multiple-testing-corrected significance. Genetically predicted sphinganine-1-phosphate levels were positively associated with POI risk; therefore, this metabolite was interpreted as a corrected-significant, risk-associated metabolite candidate. The remaining 53 exposures were considered nominal MR signals rather than definitive causal associations. Given the limited number of POI cases in the outcome GWAS, these nominal associations were interpreted as exploratory signals requiring replication in larger POI GWAS datasets. Sensitivity analyses for the 54 nominally associated exposures, including heterogeneity, horizontal pleiotropy, MR-PRESSO, leave-one-out assessment, and metabolite-level Steiger directionality assessment, are summarized in Supplementary Table S4. In this analysis, Steiger directionality was consistent with the inferred metabolite-to-POI direction for all 54 nominally associated exposures. However, this observation was interpreted cautiously because POI is a binary outcome, SNP-level FinnGen outcome sample sizes were unavailable, and the apparent uniform directionality may partly reflect limited power or instability of variance-explained estimates under the available outcome data structure.

### 3.4 Reverse MR analysis of nominally associated metabolites

**FIGURE 3:**
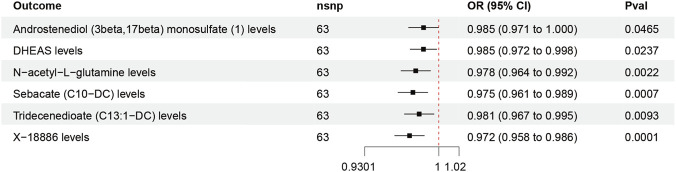
Exploratory reverse MR analysis of POI-related genetic liability and circulating metabolites. The forest plot shows nominal POI-to-metabolite associations estimated using the IVW method. Reverse MR was performed as a secondary directionality analysis and was not interpreted as definitive evidence of reverse causality. Sebacate showed discordant directionality between forward and reverse MR and was not prioritized for protective functional screening.

To further assess directionality, reverse MR was performed using POI as the exposure and nominally associated metabolites as outcomes. Six metabolites showed nominal POI-to-metabolite associations (Figure 3 and Supplementary Table S5). These findings suggest that genetic liability to POI may also be associated with alterations in circulating metabolite levels. However, because reverse MR was conducted as a secondary directionality analysis, these results were interpreted as exploratory signals rather than definitive evidence of reverse causality.

### 3.5 Direction-based prioritization of metabolites for functional screening

Because the cell-based experiments aimed to identify metabolites with potential protective activity against granulosa-cell injury, the direction and interpretability of MR associations were considered during candidate prioritization. Importantly, downstream experimental prioritization was not based solely on multiple-testing-corrected statistical significance, but also on the intended purpose of the functional screen.

The only multiple-testing-corrected metabolite, sphinganine-1-phosphate, showed a positive association with POI risk and was therefore interpreted as a corrected-significant risk-associated metabolite candidate rather than a protective intervention candidate for supplementation-based cell experiments. By contrast, N-acetyl-L-glutamine did not survive FDR or Bonferroni correction and was not interpreted as a corrected-significant MR finding. However, it belonged to the chemically defined individual metabolites showing nominal inverse associations with POI risk.

To reduce ambiguity in experimental prioritization, metabolite ratios and metabolites annotated as unknown were not selected for direct cell-based validation. After excluding these categories from the nominal inverse MR signals, 18 chemically defined individual metabolites remained and were considered as the protective candidate pool. These candidates were further evaluated according to MR direction, chemical interpretability, biological or pathway rationale, practical feasibility for *in vitro* treatment, and metabolic-class representativeness. Because biological plausibility, chemical tractability, metabolic-class representation, and experimental feasibility were incorporated into the selection process, this prioritization should be interpreted as hypothesis-generating rather than as an objective ranking of all nominally associated metabolites.

N-acetyl-L-glutamine was prioritized because it had a clear glutamine-related biochemical identity, showed a nominal inverse association with POI risk, and was biologically relevant to redox balance and mitochondrial homeostasis. Therefore, N-acetyl-L-glutamine was not selected as a statistically confirmed POI-associated metabolite, but as a functional candidate from chemically defined nominal inverse signals for experimental screening. Four additional metabolites, including 3-formylindole, DHEAS, 4-methyl-2-oxopentanoate, and sucrose, were included as comparator compounds to represent different metabolic contexts, including indole-related metabolism, steroid/endocrine-related metabolism, branched-chain amino-acid-related metabolism, and carbohydrate-related metabolism.

Metabolites showing inconsistent directions between forward and reverse MR were not prioritized as primary protective candidates. For example, Sebacate (C10-DC) showed discordant directionality between forward and reverse analyses, suggesting that its relationship with POI-related metabolic changes may be complex or bidirectional. Therefore, it was not selected for protective functional screening.

The prioritization criteria for the 18 chemically defined nominal inverse metabolites are summarized in Supplementary Table S6A, and the screening roles of the five selected metabolites are summarized in Supplementary Table S6B.

### 3.6 Experimental screening identified N-acetyl-L-glutamine as a protective candidate for further validation

**FIGURE 4:**
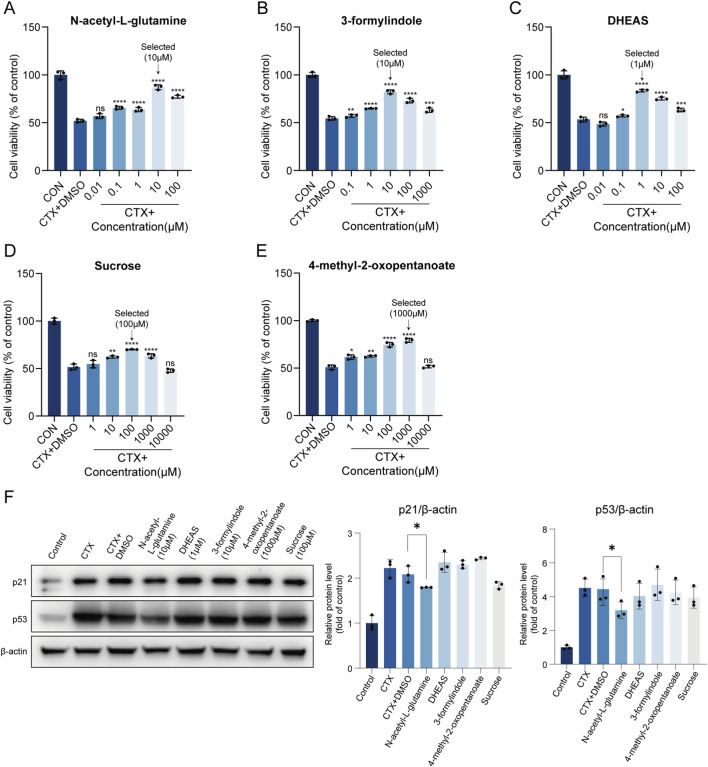
Preliminary functional screening of MR-nominated candidate metabolites in CTX-injured KGN cells. **(A–E)** CCK-8 assays were performed to evaluate cell viability responses to concentration gradients of N-acetyl-L-glutamine, 3-formylindole, DHEAS, sucrose, and 4-methyl-2-oxopentanoate under CTX-injured conditions. CTX + DMSO-treated cells were used as the vehicle model control. The selected working concentration for each metabolite is indicated by an arrow. **(F)** Representative western blots and densitometric quantification of p21 and p53 expression in CTX-injured KGN cells treated with the selected working concentrations of the five candidate metabolites. Protein levels were normalized to β-actin and expressed relative to the control group. N-acetyl-L-glutamine showed the most consistent attenuation of CTX-induced p21 and p53 upregulation among the screened metabolites and was selected for further functional validation. Data are presented as mean ± SD from three independent experiments. Statistical significance was determined by one-way ANOVA followed by Tukey’s *post hoc* test. For metabolite-treated groups, comparisons were mainly made against the CTX + DMSO vehicle group. ns, not significant; *P < 0.05; **P < 0.01; ***P < 0.001; ****P < 0.0001. The five metabolites were selected from chemically defined nominal inverse MR signals to represent different metabolic contexts and to enable preliminary functional screening.

Based on the prioritization strategy described above, five experimentally tractable metabolites were selected from the 18 chemically defined nominal inverse metabolites for preliminary functional screening: N-acetyl-L-glutamine, 3-formylindole, DHEAS, 4-methyl-2-oxopentanoate, and sucrose (Figure 4A–E). N-acetyl-L-glutamine was considered the primary functional candidate, whereas the other four metabolites were included as comparator compounds to provide metabolic-class diversity.

CCK-8 assays were first performed in CTX-injured KGN cells to determine suitable working concentrations for each compound. CTX + DMSO-treated cells were used as the vehicle-treated injury control. Across the tested concentration gradients, the candidate metabolites showed distinct viability responses, which enabled the selection of working concentrations for subsequent protein-based screening.

We next compared the effects of the five candidate metabolites, at their selected working concentrations, on CTX-induced p21 and p53 upregulation using Western blotting (Figure 4F). Compared with the control treatment, the CTX treatment increased p21 and p53 expression, and the CTX + DMSO-treated cells served as the model control for evaluating metabolite effects. Among the five screened metabolites, N-acetyl-L-glutamine showed the most consistent attenuation of p21 and p53 upregulation compared with that in the CTX + DMSO group, whereas the other metabolites exhibited weaker or less consistent effects. Therefore, N-acetyl-L-glutamine was selected for further functional validation.

### 3.7 N-acetyl-L-glutamine attenuated CTX-induced senescence, oxidative stress, and mitochondrial dysfunction in KGN cells

**FIGURE 5:**
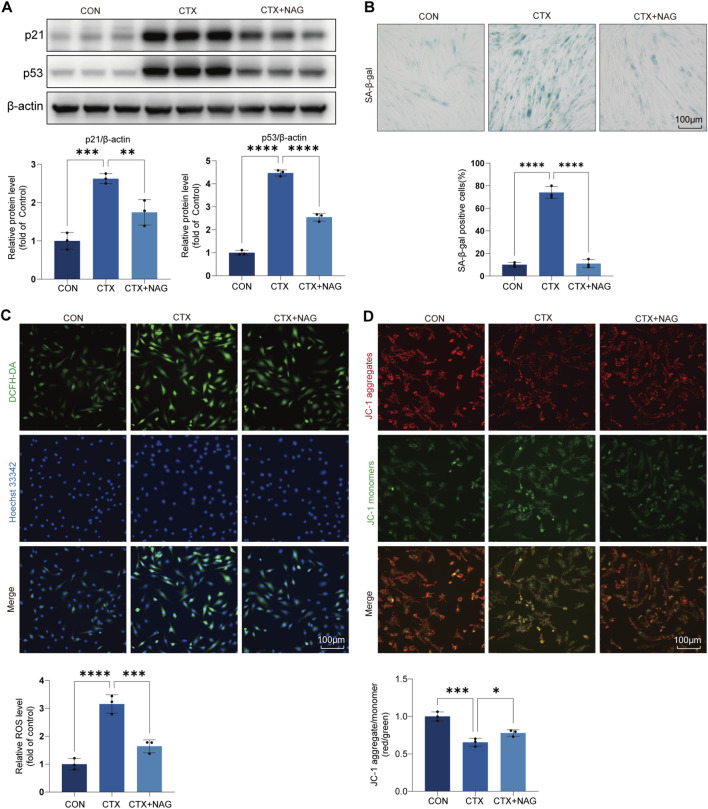
N-acetyl-L-glutamine attenuates CTX-induced senescence, oxidative stress, and mitochondrial dysfunction in KGN cells. **(A)** Representative western blots and densitometric quantification of p21 and p53 expression in KGN cells treated with CTX in the presence or absence of N-acetyl-L-glutamine. Protein levels were normalized to β-actin and expressed relative to the control group. **(B)** Representative SA-β-gal staining images and quantification of SA-β-gal-positive cells. **(C)** Representative DCFH-DA fluorescence images and quantification of intracellular ROS levels. Hoechst 33342 was used for nuclear staining. **(D)** Representative JC-1 fluorescence images and quantification of the JC-1 aggregate/monomer fluorescence ratio. A decreased aggregate/monomer ratio indicates mitochondrial membrane potential loss. N-acetyl-L-glutamine partially restored this ratio in CTX-injured KGN cells. N-acetyl-L-glutamine was used at 10 μM. Scale bars, 100 μm. Data are presented as mean ± SD from three independent experiments (n=3). Statistical significance was determined by one-way ANOVA followed by Tukey’s *post hoc* test. *P < 0.05; ***P < 0.001; ****P < 0.0001. CON, control; CTX, cyclophosphamide; NAG, N-acetyl-L-glutamine.

To further evaluate the protective effect of N-acetyl-L-glutamine, we performed functional assays in CTX-injured KGN cells. Western blot analysis revealed that CTX markedly increased p21 and p53 expression, whereas N-acetyl-L-glutamine treatment partially attenuated these changes (Figure 5A). Consistently, SA-β-gal staining revealed that CTX exposure increased the proportion of senescent cells, whereas N-acetyl-L-glutamine treatment significantly reduced the percentage of SA-β-gal-positive cells (Figure 5B).

We next evaluated intracellular ROS accumulation and mitochondrial membrane potential because oxidative stress and mitochondrial dysfunction are key features of granulosa cell injury. DCFH-DA staining revealed that CTX exposure increased intracellular ROS levels, whereas N-acetyl-L-glutamine treatment significantly reduced the ROS fluorescence intensity (Figure 5C). JC-1 staining further revealed that CTX reduced the JC-1 aggregate/monomer fluorescence ratio, indicating the loss of mitochondrial membrane potential. N-acetyl-L-glutamine partially restored this ratio, suggesting the attenuation of mitochondrial dysfunction (Figure 5D). These findings suggest that N-acetyl-L-glutamine attenuates CTX-induced injury phenotypes in granulosa-like cells by reducing senescence, oxidative stress, and mitochondrial dysfunction.

### 3.8 Protein–metabolite MR identified candidate proteins linked to N-acetyl-L-glutamine levels

**FIGURE 6:**
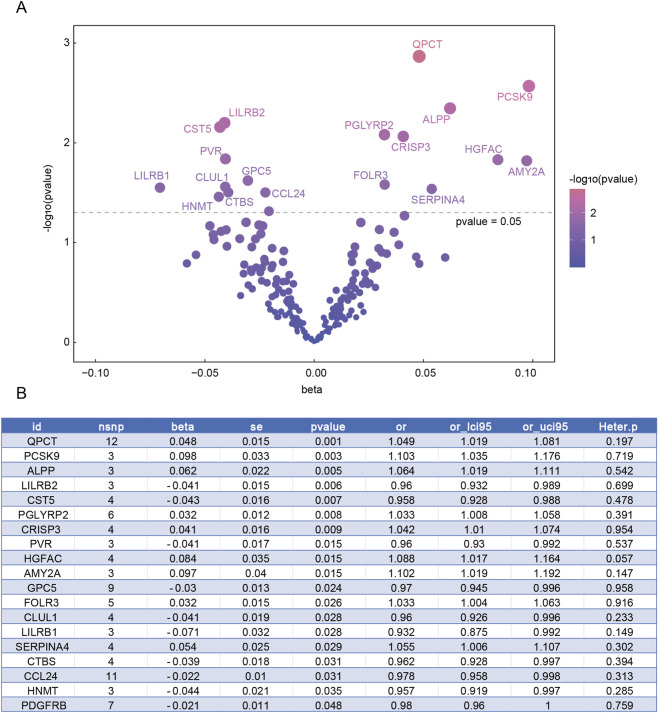
Protein–metabolite MR results for plasma proteins associated with N-acetyl-L-glutamine levels. **(A)** Volcano plot of MR effect estimates and P values. **(B)** Summary of the 19 nominally associated plasma proteins. Plasma proteins were treated as exposures and N-acetyl-L-glutamine levels as the outcome.

To explore candidate proteins genetically associated with N-acetyl-L-glutamine levels, we performed protein–metabolite MR using plasma proteins as exposures and N-acetyl-L-glutamine as the outcome. This analysis revealed 19 plasma proteins nominally associated with N-acetyl-L-glutamine levels (Figure 6), including QPCT, PCSK9, ALPP, LILRB2, CST5, PGLYRP2, CRISP3, PVR, HGFAC, AMY2A, GPC5, FOLR3, CLUL1, LILRB1, SERPINA4, CTBS, CCL24, HNMT, and PDGFRB.

In this analysis, proteins were used as exposures, and N-acetyl-L-glutamine levels were used as the outcome; therefore, these findings should be interpreted as protein–metabolite associations rather than evidence that N-acetyl-L-glutamine directly regulates these proteins.

### 3.9 Colocalization and SMR identified LILRB1 as an exploratory candidate

Among the 19 plasma proteins nominally associated with N-acetyl-L-glutamine levels, LILRB1 was further examined because it showed the highest PP.H4 value in the colocalization analysis and was also evaluated by SMR/HEIDI analysis. Colocalization analysis was performed to evaluate whether protein-associated variants and N-acetyl-L-glutamine-associated variants shared a colocalized genetic signal. LILRB1 showed the highest posterior probability for colocalization, with PP.H4 = 0.750 (Table 1), reaching the predefined threshold used for colocalization evaluation.

**TABLE 1:** Colocalization analysis of candidate proteins associated with N-acetyl-L-glutamine levels.

id	nsnps	PP.H0.abf	PP.H1.abf	PP.H2.abf	PP.H3.abf	PP.H4.abf
LILRB1	3	1.17E-08	2.50E-01	3.55E-11	6.72E-06	0.750
HGFAC	4	3.86E-05	5.94E-01	3.67E-07	5.25E-03	0.401
PCSK9	3	2.32E-07	7.30E-01	1.15E-10	9.10E-05	0.270
PVR	3	1.17E-29	8.13E-01	3.03E-33	2.38E-05	0.187
LILRB2	3	1.60E-110	9.44E-01	2.26E-114	7.74E-05	0.056
CRISP3	4	3.28E-28	9.46E-01	3.80E-32	5.54E-05	0.054
QPCT	12	6.69E-38	9.41E-01	4.94E-40	6.90E-03	0.052
CTBS	4	2.84E-05	9.49E-01	1.13E-08	3.27E-04	0.050
CST5	4	7.16E-30	9.56E-01	1.74E-33	1.88E-04	0.044
SERPINA4	4	6.12E-08	9.66E-01	1.01E-11	1.25E-04	0.034
PGLYRP2	6	3.69E-97	9.70E-01	8.06E-101	1.82E-04	0.029
HNMT	3	1.34E-52	9.71E-01	3.23E-56	2.05E-04	0.029
FOLR3	5	2.56E-63	9.73E-01	3.42E-67	1.03E-04	0.027
CCL24	12	3.71E-45	9.80E-01	1.73E-48	4.36E-04	0.019
AMY2A	3	1.15E-06	9.77E-01	5.40E-09	4.56E-03	0.019
ALPP	3	3.83E-12	9.81E-01	7.86E-16	1.83E-04	0.018
PDGFRB	7	1.02E-199	9.84E-01	2.41E-203	2.18E-04	0.016
GPC5	9	1.18E-35	9.89E-01	3.16E-39	2.55E-04	0.011
CLUL1	4	1.03E-17	9.91E-01	6.41E-21	6.08E-04	0.008

PP.H4 represents the posterior probability that the protein-related QTL signal and N-acetyl-L-glutamine-related signal share a colocalized genetic signal. PP.H4 ≥ 0.75 was considered a predefined threshold for colocalization evaluation. However, colocalization signals based on sparse variant coverage should be interpreted cautiously. The LILRB1 signal reached this threshold but was based on only three SNPs; therefore, LILRB1 was considered an exploratory candidate warranting further investigation rather than a robust colocalized signal.

**FIGURE 7:**
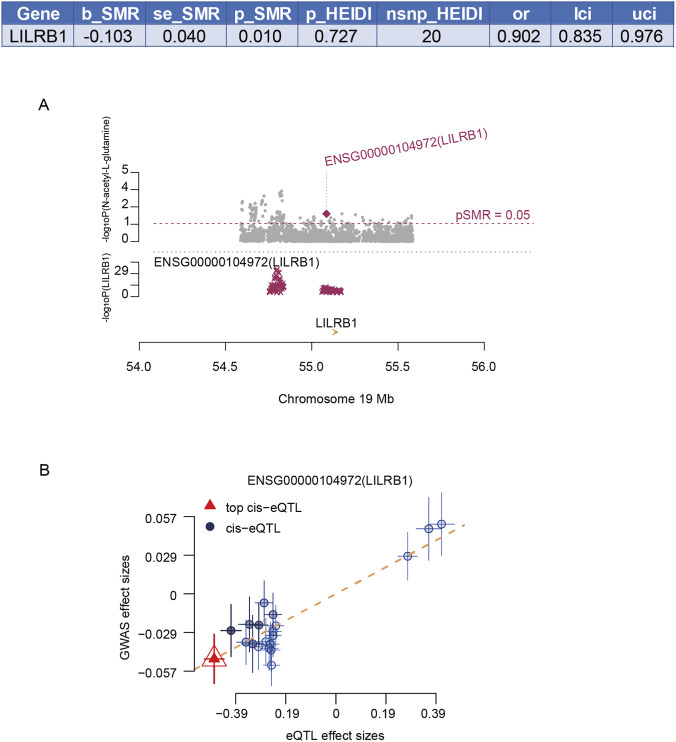
SMR and HEIDI analyses of LILRB1 and N-acetyl-L-glutamine levels. **(A)** Regional association plot showing the SMR signal for LILRB1 and N-acetyl-L-glutamine levels at the chromosome 19 locus. The dashed line indicates the nominal SMR significance threshold. **(B)** Effect-size plot comparing SNP effects on LILRB1 expression with SNP effects on N-acetyl-L-glutamine levels. The top cis-eQTL is highlighted in red. SMR analysis identified an association between LILRB1 and N-acetyl-L-glutamine levels, and the HEIDI test did not indicate significant heterogeneity. Because the LILRB1 colocalization result reached the predefined threshold but was based on only three SNPs, LILRB1 was interpreted as an exploratory candidate warranting further investigation. These results do not establish that LILRB1 mediates the protective effect of N-acetyl-L-glutamine in CTX-injured KGN cells.

However, this value was exactly at the threshold and the result was based on only three SNPs. Therefore, the colocalization evidence was considered limited and interpreted cautiously. SMR analysis further identified an association between LILRB1 and N-acetyl-L-glutamine levels, and the HEIDI test did not suggest heterogeneity consistent with linkage disequilibrium-driven association (Figure 7). Taken together, these analyses identified LILRB1 as an exploratory candidate protein linked to N-acetyl-L-glutamine levels that warrants further investigation. However, these findings do not establish a robust colocalized causal signal or demonstrate that LILRB1 mediates the protective effect of N-acetyl-L-glutamine in CTX-injured KGN cells.

### 3.10 Exploratory drug prediction and molecular docking of cianidanol with LILRB1

Candidate compounds related to LILRB1 were predicted using DSigDB as an exploratory analysis. Several candidate molecules were identified on the basis of the nominal P values (Table 2), but none remained significant after multiple-testing correction. Therefore, these results were interpreted as computational leads rather than validated drug-repurposing evidence.

**TABLE 2:** Results of the candidate drug prediction.

Term	P-value	Adjusted P-value	Odds ratio	Combined score	Genes	Regulation
Andriol CTD 00000567	0.010	0.052	19802	91390.798	LILRB1	UP
norgestrel CTD 00006422	0.010	0.052	19795	90670.752	LILRB1	UP
Sodium dichromate CTD 00000827	0.010	0.052	19792	90369.469	LILRB1	DOWN
pregnenolone PC3 UP	0.023	0.085	19544	73895.894	LILRB1	UP
methotrexate CTD 00006299	0.031	0.093	19381	67356.496	LILRB1	DOWN
cianidanol CTD 00005600	0.043	0.109	19130	59972.531	LILRB1	DOWN

None of the predicted compounds remained significant after multiple-testing correction. Therefore, these compounds were interpreted as exploratory computational leads rather than validated therapeutic candidates.

Among the predicted compounds, sodium dichromate and methotrexate were not prioritized because of known toxicity concerns. Cianidanol was retained as an exploratory computational lead because it was annotated in DSigDB as a LILRB1-downregulating compound and had fewer apparent toxicity concerns than sodium dichromate or methotrexate did.

**FIGURE 8:**
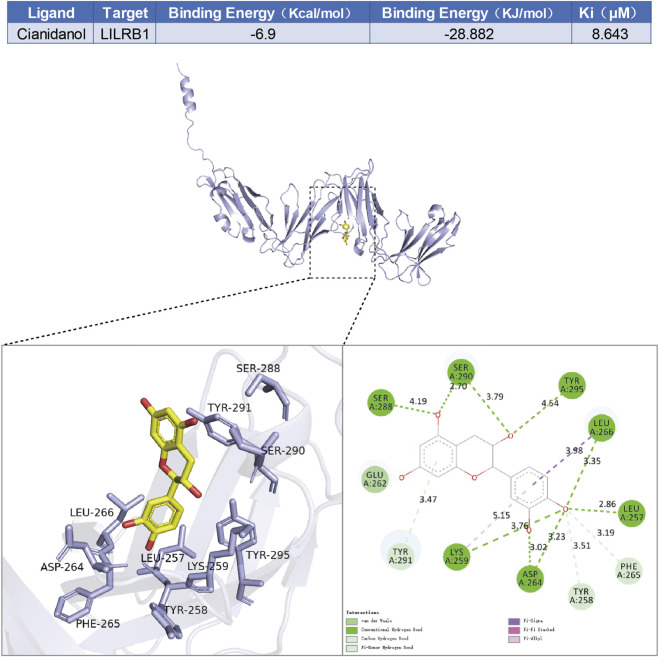
Exploratory molecular docking of cianidanol with LILRB1. Molecular docking predicted a possible binding pose between cianidanol and LILRB1, with an estimated binding energy of −6.9 kcal/mol. Docking suggests a possible physical interaction but does not establish whether cianidanol functionally activates, inhibits, or otherwise modulates LILRB1.

Molecular docking was then performed to explore the potential binding mode between cianidanol and LILRB1. Docking analysis predicted a binding pose with an estimated binding energy of −6.9 kcal/mol (Figure 8). The predicted binding region involved residues including Ser, Tyr, Leu, Lys, Asp, and Phe, and hydrogen bonding appeared to contribute to ligand binding. These results suggest a possible physical interaction between cianidanol and LILRB1; however, docking alone does not establish whether cianidanol functionally activates, inhibits, or otherwise modulates LILRB1.

## 4 Discussion

Infertility affects approximately 186 million people worldwide, and POI is an important contributor to female reproductive dysfunction (Special Programme of Research, Development, and Research Training in Human Reproduction World Health Organization, 2025). Given the marked heterogeneity of POI, identifying disease-relevant metabolic candidates may provide new insights into POI biology and potential nonhormonal intervention strategies. In this study, we integrated metabolome-wide genetic prioritization, bidirectional directionality analysis, cell-based functional screening, and exploratory downstream analyses. This framework enabled us to distinguish a multiple-testing-corrected risk-associated metabolic signal from experimentally prioritized protective candidates and to generate functional hypotheses beyond purely observational metabolomic associations.

Metabolome-wide MR analysis provides a statistically transparent framework for evaluating POI-related circulating metabolites. Sphinganine-1-phosphate was the only metabolite that survived both FDR and Bonferroni correction and was positively associated with POI risk. This finding suggests that sphingolipid-related metabolic dysregulation may be linked to POI susceptibility and may represent a risk-associated metabolic signal for future biomarker-oriented studies. In contrast, N-acetyl-L-glutamine showed a nominal inverse association with POI risk and was selected for functional screening because of its protective direction, clear biochemical identity, and experimental feasibility. Thus, these two metabolites represent distinct but complementary aspects of this study: sphinganine-1-phosphate highlights a multiple-testing-corrected risk-associated signal, whereas N-acetyl-L-glutamine represents a functionally supported protective candidate. This distinction is important because the limited number of POI cases reduces power for detecting modest associations and increases uncertainty for nominal MR signals. Therefore, the prioritization of N-acetyl-L-glutamine should be understood as functional prioritization of a chemically defined nominal inverse MR signal, rather than statistical confirmation of a metabolome-wide causal association. Because the selection process incorporated biological plausibility and experimental feasibility, it should also be viewed as hypothesis-generating rather than as an objective ranking of all candidate metabolites. Together, these findings expand current evidence linking metabolic disturbance to ovarian dysfunction and support the possibility that some circulating metabolites may be involved in POI-related biology rather than merely reflecting disease status (Liu et al., 2020; Moslehi et al., 2021; Zhou et al., 2023).

The functional screening results provided preliminary experimental evidence for the biological relevance of N-acetyl-L-glutamine. Among the five experimentally tractable metabolites tested in CTX-injured KGN cells, N-acetyl-L-glutamine showed the most consistent attenuation of CTX-induced p21 and p53 upregulation. Further assays demonstrated that N-acetyl-L-glutamine reduced the proportion of SA-β-gal-positive cells, attenuated the accumulation of ROS, and partially restored mitochondrial membrane potential. These findings indicate that N-acetyl-L-glutamine can modulate multiple injury-related programs relevant to granulosa-cell dysfunction, including senescence-like changes, oxidative stress, and mitochondrial impairment. Given that CTX-induced ovarian injury is closely related to oncofertility concerns, these results support the use of N-acetyl-L-glutamine as a biologically plausible candidate for further investigation of chemotherapy-associated ovarian damage.

The protective activity of N-acetyl-L-glutamine is also consistent with its glutamine-related biochemical nature. Glutamine is central to cellular metabolism. It serves as an important nitrogen donor for protein and nucleic acid synthesis, supports immune cell function and nitrogen balance, and sustains mitochondrial oxidative metabolism under stress conditions (Andrews & Griffiths, 2002; Cruzat et al., 2018; Yoo et al., 2020). Glutamine-related pathways can also support antioxidant defence, partly through glutathione synthesis and the limitation of oxidative damage. In addition, mitochondrial glutathione is important for maintaining mitochondrial redox homeostasis and cell survival under stress (Amores-Sánchez & Medina, 1999; Ribas et al., 2014). These properties provide a plausible metabolic explanation for the observed reduction in ROS and partial restoration of mitochondrial membrane potential after N-acetyl-L-glutamine treatment. In this context, N-acetyl-L-glutamine may help preserve granulosa cell homeostasis under injurious conditions by supporting redox balance and mitochondrial stability.

The reverse MR analysis adds another layer to the interpretation of POI-related metabolic dysregulation. Several nominal POI-to-metabolite associations were observed, suggesting that metabolic alterations in POI may not follow a strictly unidirectional model. Instead, ovarian dysfunction, hypoestrogenism, and systemic metabolic remodelling may interact in a feed-forward manner. This interpretation is consistent with the clinical view that POI is not only a reproductive disorder but also an endocrine condition accompanied by broader systemic changes. Therefore, bidirectional metabolic dysregulation may represent an important feature of POI biology and warrants further investigation in future longitudinal metabolomic studies.

The downstream protein and compound analyses were exploratory and were used to generate candidate hypotheses for future studies. LILRB1 was selected from the nominally associated proteins because it showed the highest PP.H4 value among the examined candidate proteins and was further evaluated using SMR/HEIDI analysis. However, the colocalization evidence for LILRB1 was limited because the PP.H4 value was exactly at the predefined threshold and was based on only three SNPs. Therefore, LILRB1 should be interpreted as an exploratory immune-metabolic candidate rather than as a validated mediator or therapeutic target. Its established immune-regulatory functions make it biologically relevant to POI-related metabolic remodelling, as immune activation, autoimmune disturbance, inflammation, and tissue injury have been implicated in ovarian dysfunction and POI-related pathophysiology (Chen et al., 2022; Kunicki et al., 2024). LILRB1 is broadly expressed in immune cell populations (Carenza et al., 2019; Colonna et al., 1997; Lesport et al., 2011; Lewis Marffy & McCarthy, 2020; Mori et al., 2008; Tedla et al., 2008) and is involved in cytokine release, antigen presentation, phagocytosis, cytotoxicity, and antibody-related responses(De Louche & Roghanian, 2022; Merlo et al., 2005; Naji et al., 2014; Tenca et al., 2005; Young et al., 2008). LILRB1 has also been implicated in infection, autoimmune disorders, and cancer(Abdallah et al., 2021; Deng et al., 2021; Doníz-Padilla et al., 2011; Hudson & Allen, 2016; Kuroki et al., 2005; Monsiváis-Urenda et al., 2013; Zeller et al., 2023). In parallel, cianidanol was retained as an exploratory computational lead because it was annotated as a LILRB1-downregulating compound and has been reported to have antioxidant, anti-inflammatory, and metabolic regulatory properties, including effects on insulin resistance and mitochondrial function (Wen et al., 2022). Together, these analyses suggest a preliminary immune-metabolic hypothesis involving N-acetyl-L-glutamine-related biology, LILRB1, and potential pharmacological modulation. However, whether LILRB1 acts within granulosa cells or primarily reflects immune-cell-mediated remodeling of the ovarian microenvironment remains unknown. Based on its established immune-regulatory functions, LILRB1 may be more relevant to immune-cell-mediated ovarian microenvironment remodeling than to a confirmed granulosa-cell-intrinsic pathway. Future studies should therefore examine LILRB1 expression and function in primary granulosa cells, ovarian immune-cell populations, and *in vivo* POI models.

Several limitations should be acknowledged. First, experimental validation was performed using a CTX-induced KGN cell model. Although KGN cells retain granulosa-cell-like features and are widely used in ovarian biology studies, their tumor-derived and immortalized nature limits direct extrapolation to primary granulosa cells. Because the MR analysis was based on circulating metabolites, the present study cannot determine whether N-acetyl-L-glutamine acts through systemic metabolic or immune effects, through direct exposure of granulosa cells within the follicular microenvironment, or through a combination of these mechanisms. Second, N-acetyl-L-glutamine was prioritized based on nominal MR evidence and functional screening rather than multiple-testing-corrected MR evidence. Third, although weak-instrument bias and horizontal pleiotropy were addressed using F-statistic filtering, MR sensitivity analyses, and multiple-testing correction, residual pleiotropy cannot be fully excluded. Although Steiger directionality was consistent with the inferred metabolite-to-POI direction for nominally associated exposures, this assessment should still be interpreted cautiously because the POI outcome was binary, SNP-level outcome sample sizes were not available, and the finding that all 54 exposures showed the same inferred direction may partly reflect limited Steiger test power under the available outcome data structure. Fourth, the POI GWAS included only 599 cases, which limited statistical power, particularly for detecting modest metabolite–POI associations. This limitation may increase uncertainty for nominal MR signals and may partly explain why only one metabolite remained significant after multiple-testing correction. Limited power may also contribute both to the failure to detect true metabolite–POI associations and to instability of nominal effect estimates. Therefore, the nominal associations identified in this study, including N-acetyl-L-glutamine, require replication in larger POI GWAS datasets when such resources become available. Differences between the metabolite GWAS and POI GWAS cohorts may also affect generalizability. Finally, LILRB1 and cianidanol were prioritized through exploratory downstream analyses and require direct functional validation in granulosa cells, animal models, and clinical samples. In particular, the LILRB1 colocalization result reached the predefined PP.H4 threshold but was based on only three SNPs, indicating limited robustness due to sparse variant coverage. Therefore, LILRB1 should be regarded as a hypothesis-generating candidate warranting further investigation rather than a confirmed mediator of N-acetyl-L-glutamine activity.

## 5 Conclusion

In conclusion, this study provides a metabolite-centred framework for exploring POI-related metabolic vulnerability by integrating genetic prioritization with functional screening. N-acetyl-L-glutamine was prioritized as a biologically plausible and cell-based experimentally supported protective candidate that attenuated CTX-induced senescence, oxidative stress, and mitochondrial dysfunction in granulosa-like cells. Sphinganine-1-phosphate emerged as a multiple-testing-corrected risk-associated metabolic signal, whereas LILRB1 and cianidanol generated exploratory immune-metabolic and pharmacological hypotheses for future investigation. Collectively, these findings support further investigations of metabolite candidates relevant to POI and oncofertility-related ovarian injury.

## Data Availability

The datasets analyzed in this study are publicly available. Serum metabolite GWAS summary statistics were obtained from the study by Chen et al. POI GWAS summary statistics were obtained from FinnGen release R11. Blood cis-eQTL data were obtained from the eQTLGen Consortium, and druggable gene information was obtained from Finan et al. Additional processed results are provided in the Supplementary Materials.
